# Preterm birth is associated with epigenetic programming of transgenerational hypertension in mice

**DOI:** 10.1038/s12276-020-0373-5

**Published:** 2020-01-24

**Authors:** Laurence Dumeige, Mélanie Nehlich, Say Viengchareun, Julie Perrot, Eric Pussard, Marc Lombès, Laetitia Martinerie

**Affiliations:** 1 Inserm U1185, Le Kremlin Bicêtre, 94276 France; 2 Faculté de Médecine Paris-Sud, UMR-S1185, Université Paris-Sud, Université Paris-Saclay, UMR-S 1185, Le Kremlin Bicêtre, 94276 France; 3 Service de Génétique Moléculaire, Pharmacogénétique et Hormonologie, Hôpital de Bicêtre, Assistance Publique-Hôpitaux de Paris, Le Kremlin Bicêtre, 94276 France; 4 UMS 32, Institut Biomédical de Bicêtre, Le Kremlin Bicêtre, 94276 France; 5 PremUp Foundation, Paris, 75005 France; 6 Service d’Endocrinologie et de Diabétologie Pédiatrique, Hôpital Robert Debré, Assistance Publique-Hôpitaux de Paris, Paris, 75019 France; 7 Faculté de Médecine Paris-Diderot, Université de Paris, Paris, 75019 France

**Keywords:** Pathogenesis, Hypertension

## Abstract

Renal and cardiovascular complications of prematurity are well established, notably the development of hypertension in adulthood. However, the underlying molecular mechanisms remain poorly understood. Our objective was to investigate the impact of prematurity on the ontogenesis of renal corticosteroid pathways, to evaluate its implication in perinatal renal complications and in the emergence of hypertension in adulthood. Swiss CD1 pregnant mice were injected with lipopolysaccharides at 18 days of gestation (E18) to induce prematurity at E18.5. Pups were sacrificed at birth, 7 days and 6 months of life. Second (F2) and third (F3) generations, established by mating prematurely born adult females with wild-type males, were also analyzed. Former preterm males developed hypertension at M6 (*P* < 0.0001). We found robust activation of renal corticosteroid target gene transcription at birth in preterm mice (*αENaC* (+45%), *Gilz* (+85%)), independent of any change in mineralocorticoid or glucocorticoid receptor expression. The offspring of the preterm group displayed increased blood pressure in F2 and F3, associated with increased renal Gilz mRNA expression, despite similar MR or GR expression and plasma corticosteroid levels measured by LC-MS/MS. Gilz promoter methylation measured by methylated DNA immunoprecipitation-qPCR was reduced with a negative correlation between methylation and expression (*P* = 0.0106). Our study demonstrates prematurity-related alterations in renal corticosteroid signaling pathways, with transgenerational inheritance of blood pressure dysregulation and epigenetic Gilz regulation up to the third generation. This study provides a better understanding of the molecular mechanisms involved in essential hypertension, which could partly be due to perinatal epigenetic programming from previous generations.

## High blood pressure: Exploring inheritance

A propensity towards high blood pressure may be passed down through several generations from adults who were born preterm. People who are born prematurely often suffer from kidney (renal) problems, high blood pressure and cardiovascular disease as they age. Recent research suggests adults born prematurely can pass dysregulated blood pressure to their children. Laetitia Martinerie at INSERM Unit 1185, Le Kremlin Bicêtre and Robert Debré Hospital in Paris, France, and co-workers studied generations of mice to explore how epigenetic alterations, DNA modifications that do not change the DNA code, affect blood pressure from birth through to adulthood. The team identified tissue-specific alterations in renal signaling pathways in premature mice. They also traced the associated overexpression of a gene called *Gilz*, known to play a role in blood pressure maintenance, through second and third generation mice born to the first generation preterms.

## Introduction

Prematurity is associated with various complications due to organ immaturity and impairment of physiologic organogenesis. Notably, in humans, preterm birth interrupts normal kidney organogenesis, resulting in low nephron endowment^1^, development of abnormal glomeruli^2^, and glomerular, tubulointerstitial and vascular damage, independent of nephron number^3^. In addition, kidneys of preterm neonates are immature, especially regarding tubular function and ion transport. Indeed, premature infants experience massive water and sodium losses during the first weeks of life^4^ that often challenge neonatologists to maintain a positive sodium balance^5^. Renal developmental alterations may also impact renal structure and function until adulthood and induce compensatory glomerulomegaly, renin-angiotensin-aldosterone system (RAAS) activation and glomerulosclerosis, according to the Brenner hypothesis^6^. Former preterm infants are indeed prone to developing hypertension, as early as in adolescence^7^, which leads to a global cardiovascular risk increase in this population. Interestingly, clinical evidence suggests a transmission of dysregulated blood pressure to offspring of adults born moderately preterm^8^, raising the hypothesis of a developmental programming of hypertension in this population, as it has already been suggested for essential hypertension^9^.

Developmental programming of health and diseases is defined as the early events occurring during critical periods of development that trigger permanent physiological changes responsible for future metabolic or cardiovascular diseases^10^. The suggested molecular mechanisms involved are epigenetic modifications of DNA, such as methylation of CG dinucleotide or acetylation of histones at the gene promoter level, regulating the accessibility to chromatin and the transcription of these genes^11,12^. This has been described for nuclear receptors such as GR in the brain or kidney in response to maternal stress or low-protein diet during pregnancy^13^. However, little is known about the epigenetic alterations of mineralocorticoid signaling pathways, a key regulator of blood pressure throughout life, that may be induced by prematurity.

The renal mineralocorticoid signaling pathway is indeed involved in sodium and water homeostasis through actions in the distal nephron. Aldosterone, the main mineralocorticoid hormone, is secreted by the adrenal cortex and binds to the mineralocorticoid receptor (MR), a nuclear receptor, acting as a hormone-dependent transcription factor in target cells. The MR-aldosterone complex dimerizes and interacts with mineralocorticoid response elements on DNA, allowing transcription of many target genes^14^ involved in water and sodium homeostasis, such as the alpha subunit of sodium epithelium channel (*αENaC*), serum and glucocorticoid-regulated kinase 1 (*Sgk1*) and glucocorticoid-induced leucine zipper (*Gilz*). The glucocorticoid and mineralocorticoid signaling pathways are extremely intricate in the kidney, since they share several ligands, coactivators and target genes^14^. In particular, glucocorticoid receptor (GR) activation by cortisol in humans or corticosterone in mice also regulates the transcription of *αENaC*, *Sgk1* and *Gilz*. Moreover, cortisol and corticosterone are able to bind MR. To avoid inappropriate activation of the mineralocorticoid signaling pathway by glucocorticoids in most epithelial cells, the 11β-hydroxysteroid-dehydrogenase type 2 (11βHSD2) enzyme catabolizes cortisol into cortisone (or corticosterone into 11-dehydrocorticosterone in rodents), which is unable to bind the MR^14^.

During the perinatal period, neonates experience physiological weight loss related to a low renal MR expression^15^, causing sodium and water urinary losses^3^. In contrast, GR expression in distal convoluted tubules starts early during embryogenesis and remains stable during the postnatal period. Thus, the renal MR/GR balance at birth favors the glucocorticoid pathway, which may be important for physiological maturation processes. In preterm neonates, tubular transport insufficiency is exacerbated, which could partly be explained by low aldosterone secretion of the immature adrenal cortex^16^. However, the expression of the mineralocorticoid signaling pathway and its activation in the kidneys of preterm infants has never been studied to date.

Our hypothesis is that prematurity could alter the MR/GR balance at birth and may thus induce short-term and long-term effects on the programming of corticosteroid signaling pathways involved in water and sodium reabsorption. Thus, we aimed to study in a murine model of prematurity the impact of preterm birth on the expression of corticosteroid signaling pathways at birth and into adulthood and its role in the development of early and potentially transmitted hypertension.

## Materials and methods

### Generation of preterm mice

**Fig. 1:**
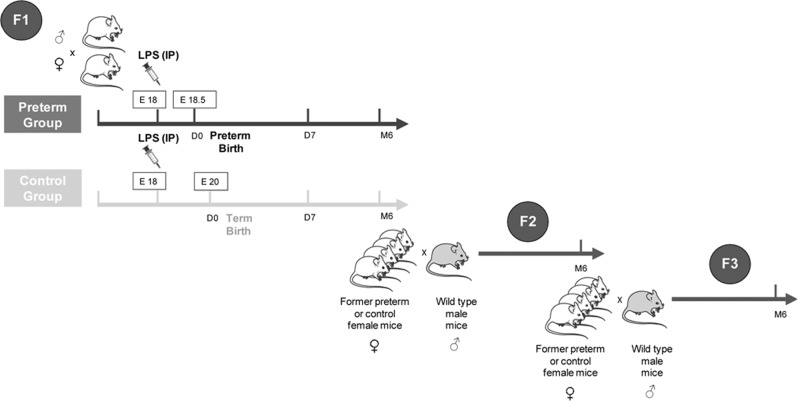
Murine model of prematurity. Preterm birth was induced by an intraperitoneal (IP) injection of 30 µg of O111:B4 lipopolysaccharides (LPS) performed at 18 days of gestation (E18) in female mice. Offspring of injected mice, when LPS did not trigger preterm birth, were used as a control to exclude intrinsic LPS effects. Preterm birth occurred at 18.5 days of gestation (E18.5) in the preterm group and at 20 days of gestation (E20) in the control group. Mice were investigated at various developmental stages (E18.5 fetuses in the control group, first day of life (D0), 7th day of life (D7), 6 postnatal months (M6)) in the first generation. By mating prematurely born adult female mice with wild-type male mice, we generated a second (F2) and a third (F3) generation. Mice were investigated at M6 in the F2 and F3 generations.

Swiss CD1 female mice (purchased from Janvier laboratories, Le Genest St. Isle, France) were mated with male mice of mixed genetic background (B6D2F1). CD1 females were chosen for their good breeding performance and for having large litters. Males of mixed genetic background (B6D2F1) were chosen to generate pups from a mixed genetic background, minimizing genetic susceptibility to hypertension. Preterm birth was induced by an intraperitoneal injection of 30 µg of O111:B4 lipopolysaccharides (LPS) (Sigma-Aldrich) performed at 18 days of gestation in female mice (Fig. 1). Birth occurred at approximately 18.5 days of gestation (E18.5) in these conditions. Offspring of injected mice, when LPS did not trigger preterm birth, were used as a control to exclude intrinsic LPS effects. In a second control group, we injected pregnant mice at 18 days gestation with PBS, which did not affect the timing of birth. However, to avoid any potential bias in the interpretation of our results that may have been induced by inflammation (secondary to LPS administration), we chose to present in this manuscript only the results obtained with the control group that received LPS, when it did not trigger preterm birth, because it seemed more relevant to us to focus on the proper effect of prematurity rather than that of inflammation, which will be the focus of another study. Litters were limited to 6 pups at birth to avoid weight changes related to litter size, which can independently program the emergence of cardiovascular diseases in adulthood. Mice were sacrificed at different developmental stages: fetuses at E18.5 in the control group, at birth (D0), at postnatal day 7 (D7), and at 6 months of age (M6). At least 6 animals were sacrificed at E18.5, D0 and D7, including males and females. At M6, six males and six females were sacrificed to evaluate potential sexual dimorphism. The body weight of each animal and the weight of the right kidney were measured. Arterial blood pressure was measured in nonanesthetized animals at M6 (see below). One kidney was fixed in buffered formol for histology. One lung, the right hemisphere of the brain and the second kidney were snap-frozen in dry ice for RT-qPCR analyses. Blood samples were also collected at sacrifice at M6 in EDTA (ethylenediaminetetraacetic acid)-containing tubes and processed for aldosterone and corticosterone measurements by LC-MSMS^17^. To generate a second generation (F2), former preterm or control female mice were mated with males of mixed genetic background (B6D2F1) at four months of life. Subsequently, F2 females of each group were mated with wild-type males to generate a third generation (F3). Arterial blood pressure was measured in six males of the F2 and F3 generations at M6 prior to sacrifice. Blood and organ samples were collected and treated with the same experimental methodology as the first generation. Mice were housed and handled according to the National Institute of Health Guidelines. The study was approved by the local and national ethics committee CEEA 26 (APAPHIS#20058).

### Blood pressure measurements

Systolic blood pressure (SBP) measurements were conducted in the animal facility of the FRIM (Fédération de Recherche en Imagerie Multi-Modalité, Paris-Diderot University, France) by tail-cuff plethysmography in trained and nonanesthetized animals as previously described^18^. Briefly, animals were acclimatized for at least 5 days before SBP measurements. Mice were restrained for less than 10 min in a clear plastic tube, and the cuff was placed on the tail and inflated to 200 mmHg. The reappearance of a pulse during deflation of the cuff was used to determine SBP. Heart rate (HR) was derived from the pulse to pulse interval. At least six recordings of SBP were measured per day for five consecutive days. The first 2 days were used for animal acclimatization. The results are expressed as the mean ± SEM of at least six measurements of SBP for each mouse of each sex per day during the last 3 days of measurements.

### Nephron number evaluation

For each animal, one formol-fixed kidney was embedded in paraffin and cut into 4-µm sections with a microtome for histologic examination. Two to three renal sections on one slide were hematoxylin-eosin stained. Stained slides were scanned using a Panoramic 250 Flash Slide Scanner (3DHISTECH Ltd. Hungary) system, with a 40× Plan-Apochromat objective and a 1.6× camera adapter magnification, to obtain high-resolution images (0.122 µm/pixel). Quantification of nephron number was performed manually by two independent examiners. Each glomerulus was annotated to avoid double counting. The glomerular density was defined as the nephron number normalized by the renal section area. The results are expressed as the mean ± SEM of the glomerular density on three different sections of the same kidney.

### Reverse transcription quantitative PCR

Total RNA was extracted from frozen samples using TRIzol reagent (Life Technologies, Villebon-sur-Yvette, France) according to the manufacturer’s recommendations. After DNAse treatment (Biolabs), 1 µg of RNA was reverse-transcribed using the High Capacity cDNA Reverse Transcription Kit (Life Technologies). Samples, 10-fold diluted, were used for quantitative PCR using the Fast SYBR Green Master Mix (Applied Biosystems) containing 300 nM of specific primers. Primer sequences are presented in Table 1. Quantitative PCR was carried out on a Quant Studio 6 Flex Real-Time PCR System (Thermo Fisher). The reaction parameters were as follows: 95 °C for 20 s, followed by 40 cycles at 95 °C for 1 s, and 60 °C for 20 s. Samples were amplified in duplicate from 2 to 3 independent reverse transcriptions. In M6 mice, we used the geometric mean of three internal control genes (*Beta-actin*, *ribosomal protein lateral stalk subunit P0 (36B4)* and *Hypoxanthine phosporibosyl-transferase 1 (HPRT1))* to normalize gene expression, using geNorm software as already described^19^. In E18.5 fetuses, D0 and D7 mice, ribosomal r18S RNA was used as a housekeeping gene, since all other normalization genes varied during renal development. The primer sequences for these internal control genes are presented in Table 1. The relative expression of each gene is expressed as the ratio of attomoles of specific gene per geometric average of control gene expression as determined by geNorm (see above) or femtomole of r18S in pups. The final results represent relative expression normalized to that obtained in samples from control mice at each age, which was arbitrarily set at one.

**Table 1:** Primers used to determine the relative expression of corticosteroid signaling pathway genes and housekeeping genes by RT-qPCR and to quantify DNA immunoprecipitation of Gilz by MeDIP.

	Name	Forward primer	Reverse primer
Gene expression (RT-qPCR)	36B4	AGCGCGTCCCATTGTCTGT	GGGCAGCAGTGGTGGCAGCAGC
	βactin	AAGTACCCCATTGAACATGCGA	CATCTTTTCACGGTTGGCCTTA
	HPRT1	AGACTTGCTCGAGATGTCATGAAG	AATCCAGCAGGTCAGCAAAGA
	18s	CCGTGCCCTTTGTACACACC	CGATCCGAGGGCCTCA
	Nr3C2	ATGGAAACCACACGGTGACCT	AGCCTTATCTCCACACACCAAG
	Nr3C1	TTCTGTTCATGGCGTGAGTACC	CCCTTGGCACCTATTCCAGTT
	*α*ENaC	GGAGTCGAAAATCGGCTTCC	TAGAGCAGGCGAGGTGTCG
	Sgk1	TCACTTCTCATTCCAGACCGC	ATAGCCCAAGGCACTGGCTA
	Gilz	CTGCTGTGGAGTTTGTGACATACTAG	CCAGGCAGGCACTTCTAAGCT
Methylation DNA immunoprecipitation-qPCR	Gilz Primer 1	GAAAACTCAGCCCTTGCTATGG	AAAGCCAAGCAAACCAAACAA
	Gilz Primer 2	CCACTTTCCTGCCCAACAAA	GAAGGAGGGAAGCAAGAAGACA
	Gilz Primer 3	CCCTGTGTTTTGCTGGCAATA	ACACTTGAAGCATTGTGTACCACAT
	Gilz Primer 4	TTGCACAGGACACAAGAATATATATGAT	GCTCAAAATAGTTGCACGAAACC
	Gilz Primer 5	GACTTGTCTAAGTATGGGTTGAATCTACA	GGAGCAAGCTTATACCAGGAAGTT
	Gilz Primer 6	CCCATAGTTAGTATGTCATTGATGGAA	CCACGAGGTTGCATTGAATAATAA
	Gilz Primer 7	CCCACCATCTCCCTTGGAAT	CCCTCTGCCACCTAGAGCTTT
	Gilz Primer 8	CAGATAACACTCCCGACGACCTA	TGCCAACCTCTGGACATTTTAA
	Gilz Primer 9	ATTCCTTTTTCTGCCCATGCT	AAAGAAGGCGGCATCTAAGACTT
	Gilz Primer 10	GGATGGAGGTTCTCTTTGGATTC	CGTGCTGATAACAGCTCCATCT
	Gilz Primer 11	GAGGTAGCTCAGCGGCAAGA	ACTGTACCACATGAGTGCCTTGTT
	Gilz Primer 12	AGTTGGCTGGAGAAAGTGAAGAA	GGGCGGTACTGCATTTAAAAGT

### Methylated DNA immunoprecipitation (MeDIP)

**Fig. 2:**
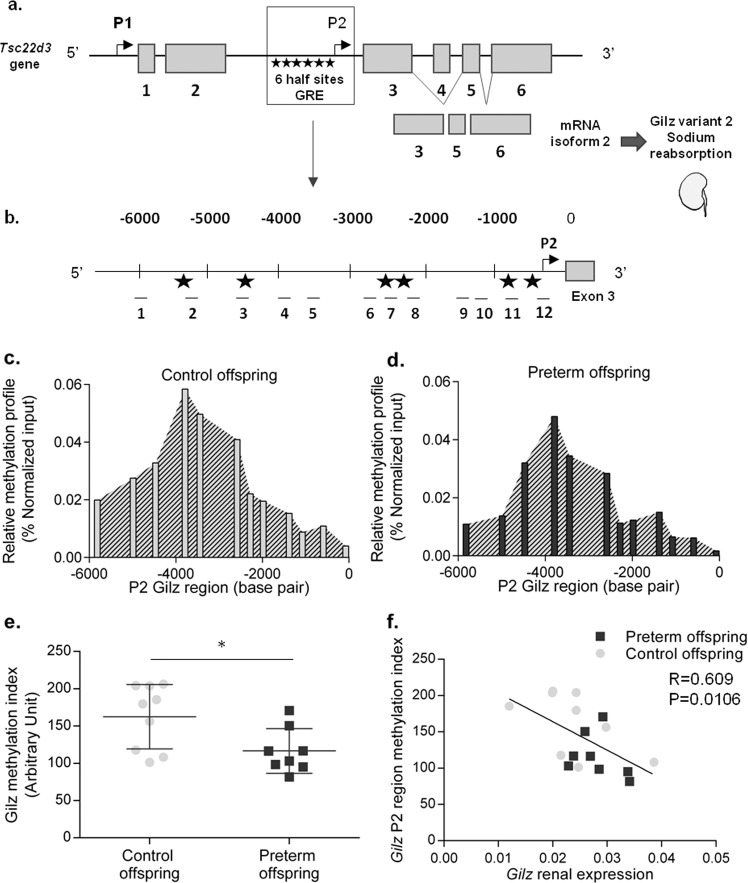
Epigenetic regulation of the Gilz gene (*Tsc22d3)* by DNA methylation in the second (F2) and third (F3) generations. Genomic structure of the *Tsc22d3* mouse gene (**a**). Each gray box represents exons of the *Tsc22D3* gene. Black arrows represent the 2 promoters regulating the transcription of the Gilz isoforms, named P1 and P2. The region upstream of P2 contains 6 half-site glucocorticoid responsive elements (GREs) represented by black stars. The alternative splicing of exon 3 allows the transcription of Gilz isoform 2, translated as protein variant 2, which is responsible for water and sodium reabsorption in the kidney. We focused on the region upstream of exon 3, regulating the transcription of Gilz’s isoform 2 (**b**). Zero has been arbitrarily defined as the first base of exon 3. The 6 GRE half-sites are represented as described above, as well as the 12 pairs of primers used to amplify the P2 region (see below). Relative methylation profile of control (**c**) and preterm (**d**) male offspring at 6 months of age (M6). Methylation profiles were determined using amplification of methylated DNA with 12 pairs of primers in the region upstream of exon 3. Methylated DNA has been immunoprecipitated by a MeDIP technique; the results presented are the mean of the percent of methylated DNA compared to the input, normalized to the *TSH2B*-positive control gene for each primer pair, represented according to its position (in base pairs) in the P2 region. *Gilz* methylation index in control and preterm male offspring at M6 (**e**) in arbitrary units. The *Gilz* methylation index was determined by calculating the area under the curve (AUC) from the methylation profiles for each mouse. Each mouse in the control group is represented by a gray dot, and each mouse in the premature group is represented by a black square, with mean and SD for each group. Nonparametric Mann–Whitney *U-*tests. Correlations between the *Gilz* methylation index and *Gilz* renal mRNA expression in the F2 and the F3 generations (**f**) were obtained by Spearman regression analysis. MeDIP was performed by pooling samples from the F2 and F3 generations (after ensuring that the results were consistent in both generations), with *n* = 8 in the preterm group and *n* = 9 in the control group. *P < 0.05, **P < 0.01, ***P < 0.001.

MeDIP of candidate genes that were differentially methylated between preterm and control offspring mice was performed with the MagMeDIP kit (Diagenode, Seraing, Belgium). DNA was extracted from frozen samples using TRIzol reagent according to the manufacturer’s recommendations for samples of the F2 and F3 generation. Each DNA sample was sheared by sonication for ten cycles with 30 s on and 30 s off at 4 °C using the Bioruptor PICO sonicator (Diagenode). RNAse treatment was performed, followed by phenol/chloroform extraction, ethanol precipitation and elution in TE buffer (10 mM Tris-HCl, 1 mM EDTA, pH 7.4). The DNA shearing efficiency was confirmed by electrophoresis on a 2% agarose gel, as demonstrated by 150 and 600 bp DNA fragments for all samples. DNA (1.1 µg) was added to the IP incubation mix and denatured at 95 °C for 3 min. One-tenth of the DNA sample was set aside at 4 °C for input. The rest (IP sample) was incubated with magnetic beads and 5-methylcytosine antibody overnight at 4 °C with mixing on a rotative wheel. Beads were rinsed 4 times with bead wash buffer, resuspended in DNA isolation buffer with 0.01% proteinase K and incubated for 15 min at 55 °C and 15 min at 100 °C. IP and input samples were amplified using qPCR. We decided to amplify the promoter region upstream of exon 3 (promoter P2), which regulates the transcription of Gilz’s isoform 2 and contains six half-site glucocorticoid response elements (GRE). Gilz isoform 2 is translated into Gilz variant 2, which is known to be responsible for sodium reabsorption in the kidney (Fig. 2). Twelve pairs of primers were designed to study 6000 base pairs, encompassing P2 and the beginning of exon 3, identified as associated with histone 3 acetylation on lysine 27 (mark associated with activation of gene transcription) both in mice and humans according to the University of California Santa Cruz (UCSC) genome browser. Primer sequences are presented in Table 1. Quantitative PCR was carried out on a Quant Studio 6 Flex Real-Time PCR System (Thermo Fisher), and amplification parameters were 95 °C for 60 s, followed by 40 cycles at 95 °C for one second and 60 °C for 45 s. Methylation quantification was calculated from qPCR data and reported as the recovery of starting material: %(meDNA-IP/Total input) = 2^[(Ct(10%input)-3.32) − Ct(meDNA-IP)] × 100%. The results were normalized to positive control testis-specific histone H2B (*TSH2B*) methylation for each sample to exclude variations in MeDIP efficiency. Glyceraldehyde-3-phosphate dehydrogenase (*GAPDH*) methylation was used as a negative control for each sample. Primers for *TSH2B* and *GAPDH* amplification were provided by Diagenode. The results obtained for the 12 amplified regions for each animal were integrated to determine a methylation profile, and the area under the curve (AUC) was evaluated and considered as a methylation index for region P2.

### Statistical analyses

Statistical analyses were performed using nonparametric Mann–Whitney *U-*tests to compare two parameters and using a nonparametric Kruskal–Wallis test to compare three parameters (GraphPad Prism 6, GraphPad software, San Diego, USA). The correlation between two parameters was obtained by Spearman regression analysis with a significant threshold set at 0.05.

## Results

### Clinical characteristics of premature mice

**Fig. 3:**
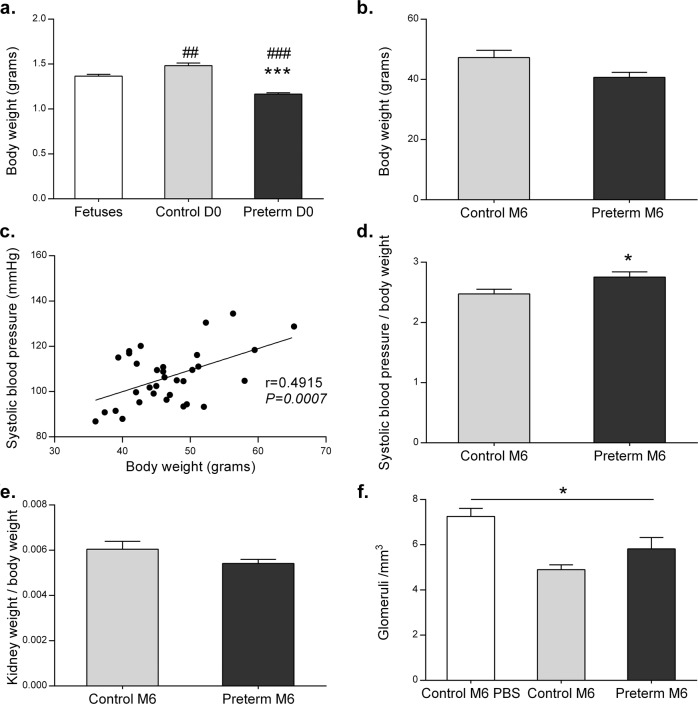
Clinical characteristics of premature mice. Birth body weight of premature or control mice, compared to E18.5 fetuses, expressed in grams, as the means ± SEM (fetuses *n* = 6, preterm D0 *n* = 14, control D0 *n* = 17) (**a**). Body weight of 6-month (M6)-old former premature males and control mice, expressed in grams, as the means ± SEM (*n* = 8 in each group) (**b**). Positive correlation between systolic blood pressure and body weight in M6 mice, obtained by Spearman regression analysis (**c**). Systolic blood pressure normalized to body weight ratio in former premature and control M6 male mice (*n* = 6 in each group) (**d**). Kidney weight to total body weight ratio in former premature and control M6 male mice (*n* = 6 in each group) (**e**). Glomerular density per mm³ measured in renal sections of former preterm and control M6 male mice (*n* = 3 in the control PBS group, *n* = 5 in the control LPS group, *n* = 4 in the preterm group) (**f**). *P < 0.05, **P < 0.01, ***P < 0.001 (compared to the control group); ^#^*P* < 0.05, ^##^*P* < 0.01, ^###^*P* < 0.005 (compared to the fetus group). Nonparametric Mann–Whitney *U-*tests or Kruskal–Wallis test for comparison between 2 or 3 parameters, respectively.

LPS injection induced premature delivery in 70% of cases. Premature mice exhibited maladaptation at birth, with a high mortality rate. In LPS-injected mice, 30% of pups were stillborn, and 35% died during the first hours of life. As anticipated, premature mice presented lower birth body weight (BW) than control newborn mice (1.17 ± 0.05 vs. 1.48 ± 0.13 g, *P* < 0.0001) but also fetuses of the same age (1.17 ± 0.05 vs. 1.37 ± 0.07, *P* < 0.0001) (Fig. 3a). This growth retardation normalized during the first weeks of life, and the BW at M6 was not significantly different between the control and premature groups (Fig. 3b). Considering interindividual disparities in BW between mice at M6 and because a positive correlation between blood pressure and BW was found in control mice in two independent experiments (*r* = 0.4915, *P* = 0.0007) (Fig. 3c), the arterial blood pressure of each mouse was normalized to BW. Indeed, former premature male mice at M6 presented increased arterial blood pressure compared to control mice (121 ± 14.15 mmHg vs. 114 ± 7.98 mmHg), which was also confirmed when blood pressure was corrected with BW (2.75 ± 0.08 vs. 2.53 ± 0.08, *P* = 0.0342) (Fig. 3d). This was not explained by a difference in kidney weight or glomerular density between the groups (Fig. 3e, f, Supplemental Fig. S1). However, we found a low nephron number in both groups compared to the second control group (PBS) that had not been subjected to LPS (*P* = 0.0124).

### F1 premature mice present with early modifications in the renal corticosteroid signaling pathways

**Fig. 4:**
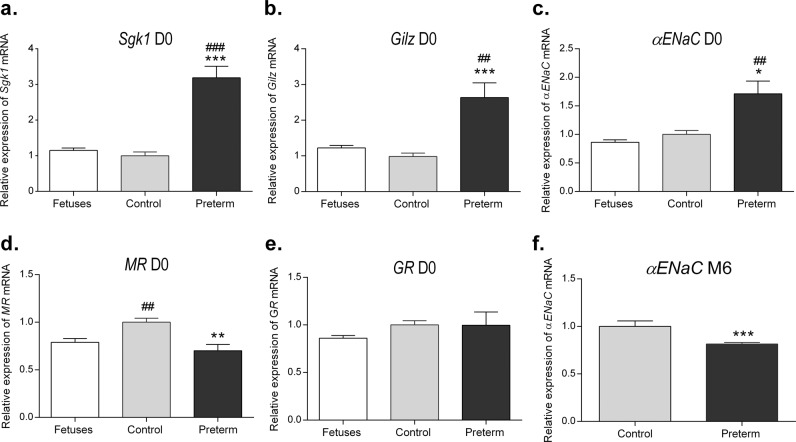
Expression of major players in renal corticosteroid signaling pathways in premature mice compared to controls and fetuses. Relative renal mRNA expression of *Sgk1* (**a**)*, Gilz* (**b**)*, αENaC* (**c**), *MR* (**d**), and *GR* (**e**) at birth. Relative renal *αENaC* mRNA expression (**f**) in former premature and control male mice at 6 months of age (M6). Relative mRNA expression in mice was determined using reverse transcription-quantitative PCR (RT-qPCR). The results are expressed as the ratio of attomoles of specific gene per attomoles of ribosomal r18S RNA in E18.5 fetuses and D0 mice, or the ratio of attomoles of specific gene per the geometric mean of three housekeeping genes (*36B4*, *beta actin* and *HPRT1*) in M6 mice, normalized to the expression of control mice, arbitrarily set at 1. *n* = 6 mice in each group. *P < 0.05, **P < 0.01, ***P < 0.001 (compared to the control group); ^#^*P* < 0.05, ^##^*P* < 0.01, ^###^*P* < 0.005 (compared to the fetus group). Nonparametric Mann–Whitney *U-*tests.

We next investigated whether prematurity had an impact on the renal expression of major players of the corticosteroid signaling pathways at birth. We found a strong activation of corticosteroid target gene mRNA expression, such as *Sgk1* (3.18 ± 0.32 vs. 0.99 ± 0.01, *P* < 0.0001), *Gilz* (2.63 ± 0.44 vs. 0.99 ± 0.09, *P* = 0.0007) and *αENaC* (1.71 ± 0.24 vs. 1.00 ± 0.07, *P* = 0.0204), in premature mice compared to expression in control mice at D0 and D7 (Fig. 4a–c, Supplemental Fig. S2). These high mRNA expression levels were not related to modifications in MR or GR abundance since *MR* mRNA expression was significantly decreased in premature mice (0.70 ± 0.06 vs. 1.00 ± 0.04, *P* = 0.0018), while *GR* expression remained unchanged (Fig. 4d, e). However, these variations were not sustained in adulthood. Indeed, *Sgk1* and *Gilz* mRNA expression levels were not significantly different at M6 between former preterm and control male mice. Furthermore, *αENaC* expression was significantly decreased in former preterm male mice compared to expression in control mice (0.81 ± 0.02 vs. 1.00 ± 0.06, *P* < 0.0001) (Fig. 4f).

### Tissue-specific alterations in perinatal corticosteroid signaling pathways in premature mice

**Fig. 5:**
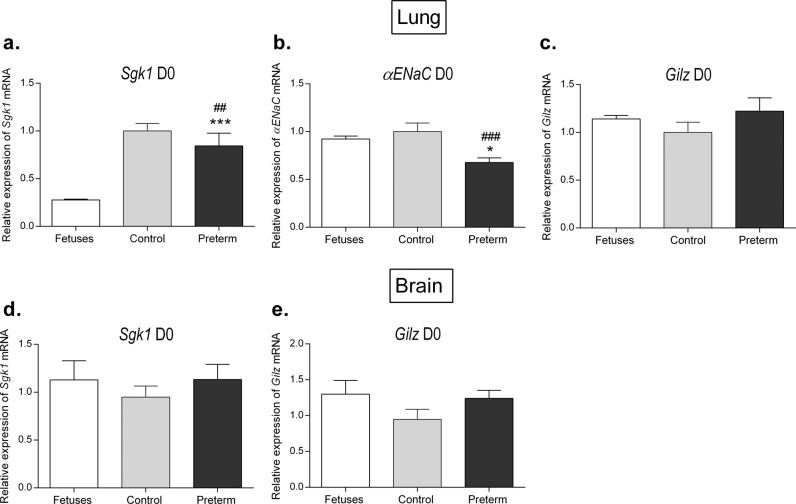
Relative pulmonary and cerebral expression of corticosteroid target genes in premature mice at birth compared to expression of controls and fetuses. Relative pulmonary mRNA expression of *Sgk1* (**a**)*, Gilz* (**b**), and *αENaC* (**c**) and cerebral mRNA expression of *Sgk1* (**d**) and *Gilz* (**e**) at birth. Relative mRNA expression in mice was determined using reverse transcription-quantitative PCR (RT-qPCR). The results are expressed as the ratio of attomoles of specific gene per attomoles of ribosomal r18S RNA, normalized to the expression of control mice, arbitrarily set at 1. *n* = 6 mice in each group. *P < 0.05, **P < 0.01, ***P < 0.001 (compared to the control group); ^#^*P* < 0.05, ^##^*P* < 0.01, ^###^*P* < 0.005 (compared to the fetus group). Nonparametric Mann–Whitney *U-*tests.

To evaluate whether modifications in corticosteroid signaling pathways at birth were tissue-specific, similar gene expression analyses were performed in the lungs and brains of preterm and control mice at birth. We found very different profiles in these organs. Indeed, *αENaC* and *Sgk1* mRNA expression was significantly reduced at birth in the lungs of premature mice compared to that of control mice (0.68 ± 0.04 vs. 1.00 ± 0.09, *P* = 0.0167; 0.84 ± 0.13 vs. 1.00 ± 0.08, *P* = 0.0045), whereas pulmonary *Gilz* mRNA expression was not significantly modified (Fig. 5a–c). No significant variation in cerebral *Sgk1* and *Gilz* mRNA expression was observed between preterm and control mice (Fig. 5d, e), indicating that prematurity impacts corticosteroid signaling pathways in neonates in a tissue-specific manner.

### Dysregulation of arterial blood pressure in F2 and F3 preterm male offspring

**Fig. 6:**
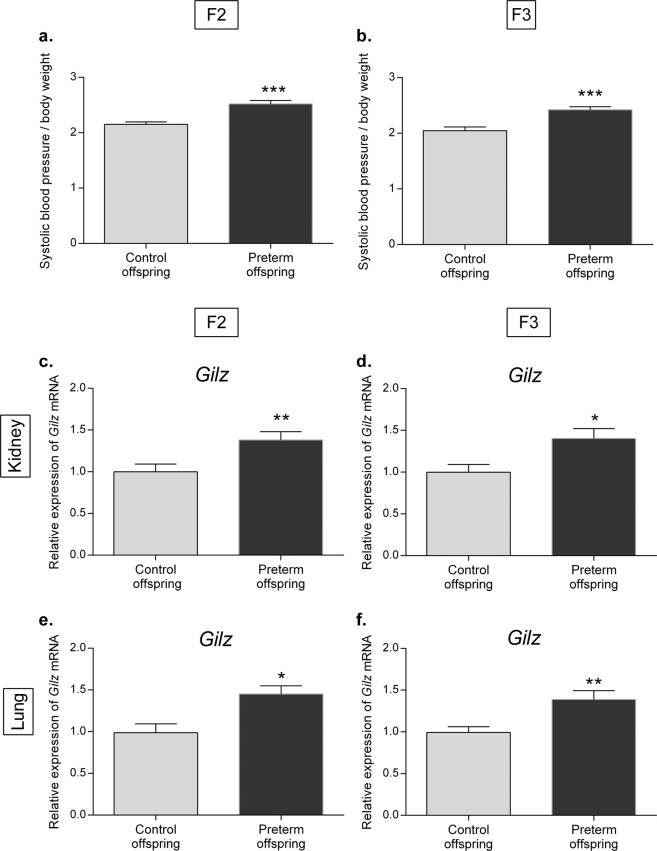
Characteristics of the offspring of premature or control male mice at 6 months of age (M6) in the second (F2) and the third (F3) generations. Systolic blood pressure to body weight ratio in former premature and control M6 male offspring in the F2 (**a**) and F3 (**b**) generations. Relative renal mRNA expression of *Gilz* in preterm and control male offspring in the F2 (**c**) and F3 (**d**) generations. Relative pulmonary mRNA expression of *Gilz* in the F2 (**e**) and F3 (**f**) generations. Relative mRNA expression in mice was determined using reverse transcription-quantitative PCR (RT-qPCR). The results are expressed as the ratio of attomoles of specific genes relative to the geometric mean of three housekeeping genes (*36B4*, *beta-actin* and *HPRT1*), normalized to the expression of control mice, arbitrarily set at 1. *n* = 6 mice in each group. *P < 0.05, **P < 0.01, ***P < 0.001. Nonparametric Mann–Whitney *U-*tests.

Arterial blood pressure was measured in male descendants of the preterm and the control group in the second (F2) and the third (F3) generations. Similar to the first generation (F1), we found a significant increase in arterial blood pressure corrected for BW in the F2 (2.52 ± 0.06 vs. 2.15 ± 0.04, *P* < 0.0001) and the F3 generations (2.42 ± 0.05 vs. 2.05 ± 0.07, *P* = 0.0005) (Fig. 6a, b), which was not associated with a difference in glomerular density between the two groups (3.44 ± 0.18 vs. 3.60 ± 0.2 glomeruli/mm^3^, *NS*).

### Increased Gilz mRNA expression in the F2 and F3 generations

We next examined whether the dysregulated blood pressure observed in F2 and F3 preterm males could be related to variations in renal corticosteroid signaling pathways. We found no difference in αENaC and Sgk1 mRNA expression in males of the F2 and F3 generations that could explain the blood pressure regulation anomalies found in these two generations (data not shown). However, interestingly, we found a significant sustained increase in renal *Gilz* mRNA expression in F2 (1.38 ± 0.10 vs. 0.99 ± 0.09, *P* = 0.0071) and F3 (1.40 ± 0.12 vs. 0.99 ± 0.09, *P* = 0.0386) descendants of the preterm group compared to expression in control offspring (Fig. 6c, d). This increase was independent of *MR* or *GR* mRNA expression, which did not differ between groups, either in F2 or in F3 (data not shown). Furthermore, we did not find any variation in plasma aldosterone concentrations (0.062 ± 0.01 ng/mL vs. 0.080 ± 0.02 ng/mL) or in plasma corticosterone concentrations (30.35 ± 3.84 ng/mL vs. 41.63 ± 7.75 ng/mL) measured by LC-MS/MS between the two groups in the F2 and F3 generations. Thus, the increased and sustained Gilz mRNA expression in the F2 and F3 generations raised the question of a potential epigenetic regulation of *Gilz*. In this context, we investigated whether this was also true in other organs, such as the lungs or brains of F2 and F3 mice. Indeed, we found a similar prematurity-induced increase in pulmonary and cerebral *Gilz* mRNA expression (1.45 ± 0.10 vs. 1.00 ± 0.10*, P* = 0.0151, and 1.39 ± 0.11 vs. 0.99 ± 0.07 *P* = 0.0025, in the lungs of the F2 and F3 generations, respectively; 1.51 ± 0.14 vs. 0.99 ± 0.07, *P* = 0.0038, 1.28 ± 0.24 vs. 0.99 ± 0.04, *P* = 0.8729, in the brains of the F2 and F3 generations, respectively (Fig. 6e, f).

### Gilz regulation by DNA methylation

We studied methylation of the P2 region upstream of exon 3 of the *Tsc22d3* gene using 12 different pairs of primers (Fig. 2a, b) in offspring of preterm and control mice at the second and third generations. We found a similar methylation profile in the offspring of premature mice compared to that of control mice (Fig. 2c, d). However, a global hypomethylation of the entire region was observed in the preterm group, with a methylation index of the P2 region that was significantly reduced (116.5 ± 10.57 vs. 162.5 ± 14.38 (arbitrary units), *P* = 0.0206) (Fig. 2e). Interestingly, we found a strong negative correlation between the expression of *Gilz* mRNA isoform 2 and the methylation index of the P2 region (*r* = −0.609 95% CI [−0.847; −0.167], *P* = 0.0106) (Fig. 2f), providing additional support for the epigenetic regulation of *Gilz* through DNA methylation of its regulatory sequences.

## Discussion

Our study provides the first evidence of alterations in renal corticosteroid pathways induced by prematurity during the perinatal period, with a transgenerational transmission of dysregulated blood pressure up to the third generation, associated with alterations in *Gilz* methylation and an increase in its expression.

The first challenge of our work was to develop a model of prematurity to study renal corticosteroid signaling from birth to adulthood. Given the difficulties in collecting human kidney samples of preterm neonates, we chose to study a murine model, especially since our group has already demonstrated a conserved ontogeny of renal corticosteroid pathways between mice and humans^15^. This LPS-induced prematurity model is a model commonly used to study parturition or the neonatal consequences of LPS, and most of these studies also find very high neonatal mortality rates^20^. However, the long-term survival of the offspring has never been a point of interest to the authors. The most important difficulty was obtaining preterm mice that could survive to adulthood, without the medical care and support that human premature infants usually receive. This has only been successfully achieved by a few groups^21^. The LPS-induced prematurity model is based on an inflammatory reaction that induces labor^22^, but inflammation may have a direct impact on corticosteroid signaling pathways expression^23^. Thus, we chose as a control group, offspring of pregnant mice that received LPS but in which no premature birth occurred, to exclude the intrinsic effect of LPS (inflammation) and focus on the proper effect of prematurity. To date, we do not know why LPS triggers early delivery in some mice but not others. However, this is not due to an absence of effect or to a differential sensitivity to LPS since all pups (born prematurely or not from LPS-injected mothers) suffer renal consequences from this exposure with a similar reduction in nephron number, suggesting a comparable effect of LPS in both groups.

In addition, premature birth was induced at 18.5 days of gestation, which is related to only mild or moderate prematurity, because this term was the earliest period leading to mouse survival under our experimental conditions. This term was also interesting with regard to the physiological evolution of the renal mineralocorticoid signaling pathway, which demonstrates a transient peak of expression at E18, as previously described^15^.

Interestingly, we demonstrated profound alterations in corticosteroid signaling pathways in preterm neonates during the perinatal period. Notably, our study highlighted decreased renal MR mRNA expression in preterm pups born at 18.5 days of gestation in comparison to fetuses of the same age^15^, suggesting that labor and delivery may induce this reduced renal MR expression. Nevertheless, the mechanisms underlying MR downregulation at birth remain unknown. Furthermore, MR expression in preterm pups was even lower than that of the control group born at term. It can be assumed, when extrapolating to human neonates, that this very weak renal MR expression could participate in the severe tubulopathy associated with water and sodium urinary losses observed in preterm neonates.

Aside from MR downregulation, we unexpectedly found a very strong activation of renal *aENaC, Sgk1*, and *Gilz* mRNA expression in preterm pups at birth. These three target genes are theoretically regulated by both MR and GR in the distal convoluted tubule and the collecting duct^24^. Due to the decrease in renal MR expression with conserved GR expression in premature pups, this transcriptional activation could be rather GR-dependent and mediated by glucocorticoids, as it has already been widely demonstrated in adult mice^25^. Unfortunately, plasma steroid levels were not measured in pregnant mice at birth, preventing confirmation of this hypothesis.

Our study identified dysregulated blood pressure as a long-term consequence of premature birth, with transgenerational inheritance up to the third generation. Several studies have already reported high blood pressure in former preterm infants, as early as in infancy or adolescence^7^. Developmental programming of cardiovascular diseases was first described by Barker et al., who proposed that early events occurred during the perinatal period, i.e., preterm birth could adversely impact organogenesis or ontogenesis of signaling pathways and be responsible for long-term cardiovascular alterations in adulthood^26^. Indeed, early hypertension has been related to a reduced number of functioning nephrons in preterms^2^ as well as in small for gestational age (SGA) children^27^. Rodrìguez et al. have shown that nephron endowment is correlated with gestational age, despite the active nephrogenesis occurring up to the 40th postnatal day to compensate for altered organogenesis in preterm children^28^. Brenner et al. proposed that this premature nephron loss could be responsible for hyperfiltration in the remaining nephrons, leading to proteinuria and glomerulosclerosis in the long term^6^. In our study, there was a low nephron endowment in both former preterm and control groups compared to wild-type mice (thus suggesting a proper effect of LPS/inflammation rather than prematurity), but this does not explain the additional increase in blood pressure in the preterm group, suggesting that there are other mechanisms involved in early hypertension.

We demonstrate for the first time a transgenerational inheritance of dysregulated blood pressure induced by preterm birth, up to the third generation. In the general population, Niiranen et al. have shown that the risk for high blood pressure crosses generations from grandparents to grandchildren, especially in cases of early-onset hypertension^9^. In a small cohort of former preterm adults and their children, Mathai et al. described subtle blood pressure abnormalities in descendants of former preterms at the age of 8 years compared to controls^8^. The pathophysiology of essential hypertension is very complex and involves a combination of several susceptibility genes, as well as environmental and epigenetic factors^29^. Interestingly, the heritability of blood pressure dysregulation across generations related to DNA polymorphisms appears to be low^30^, suggesting that epigenetic factors may be at the forefront. Molecular mechanisms involved in transgenerational transmission of diseases generally involve epigenetic modifications of DNA, including methylation of CG dinucleotides and posttranslational histone modifications of gene promoters^31^, regulating accessibility to chromatin. Such epigenetic modifications have been described for corticosteroid signaling pathway genes, notably cerebral and renal GR, in response to maternal stress or a low-protein diet during pregnancy^13,32^, as well as for 11βHSD2 and αENaC^33,34^. Herein, increased *Gilz* mRNA expression was discovered and remained sustained up to the second and third generations, likely related to a global hypomethylation of its promoter. *Tsc22d3*, the gene encoding Gilz, has already been reported to be hypomethylated in leukocytes in a cohort of adult smokers^35^ or hypermethylated in some cancers^36^ without understanding the biological significance of these variations. Gilz has multiple functions, particularly in relation to immunity, dendritic cell functions, adipogenesis, spermatogenesis and sodium reabsorption in the kidney. There are several isoforms resulting from alternative splicing, translated into different protein variants that do not have equivalent functions^37^. Notably, isoform 2 is translated into Gilz variant protein 2, which is involved in renal sodium reabsorption. Gilz acts by inhibiting the phosphorylation of Raf and the activation of the ERK pathway in renal tubular cells, which interrupts the degradation of αENaC and increases its apical membrane residency^24^. Accordingly, Gilz isoform 2 KO mice develop moderate alterations in renal sodium and water reabsorption, which are more pronounced during sodium deprivation^38^. In contrast, Gilz overexpression may lead to a moderate increase in sodium reabsorption and a subtle increase in arterial blood pressure that could predispose patients to the development of hypertension. Interestingly, Gilz has been identified as a gene associated with blood pressure variations and hypertension-related cardiac phenotype, as well as a molecular marker of thiazide response in patients with hypertension, in large transcriptome-wide analysis studies in humans^39,40^. Thus, Gilz could be considered a susceptibility gene that participates in the complex pathophysiology of hypertension. Variations in Gilz expression may thus be involved in increased blood pressure observed at the second and the third generation via a transgenerational epigenetic susceptibility to hypertension, developmentally programmed by preterm birth in the first generation.

**Fig. 7:**
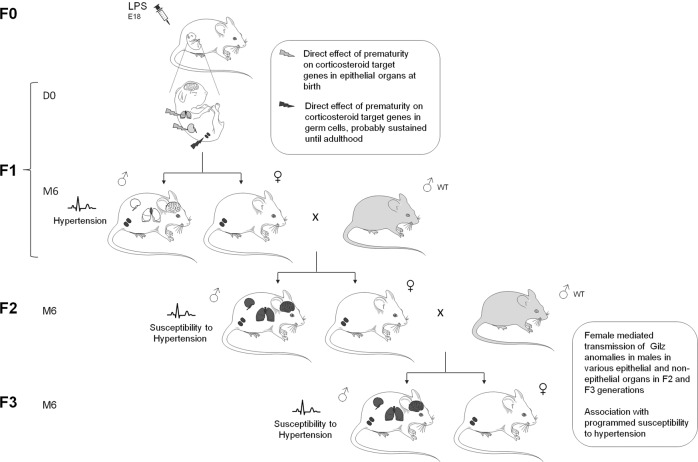
Model of the hypothetical transmission mechanisms of Gilz alterations observed from the first generation (F1) to the third generation (F3). Potential differential parallel induction of Gilz alterations in somatic cells and germ cells in F1 mice. Considering the germ cell-mediated mode of transmission, these alterations were found in all investigated organs (kidney, brain, and lung) in the F2 and F3 males. These *Gilz* alterations were associated with the transmission of dysregulated blood pressure up to the third generation after a premature birth. LPS: lipopolysaccharides, E18: 18 days of gestation, D0: first day of life, M6: 6 postnatal months, WT: wild type, F1: first generation, F2: second generation, F3: third generation.

Regulatory mechanisms underlying transgenerational transmission of a phenotype or epigenetic marks are still only partially understood, and published evidence is often incomplete^41^. Gilz promoter methylation abnormalities were only found in the second and third generations, suggesting transmission to further generations by direct alteration of the F1 germ cell epigenome that would have escaped reprogramming, as previously shown^42^ (Fig. 7). Our findings have some limitations since germ cells of F1, F2, and F3 animals were not evaluated.

In conclusion, we provide the first evidence for tissue-specific alterations in renal corticosteroid signaling pathways induced by prematurity, observed as early as in the perinatal period. These alterations could participate in the development of renal tubulopathy in premature children, consistent with hypoactivation of the renal mineralocorticoid pathway. We also demonstrate a transgenerational inheritance of dysregulated blood pressure induced by prematurity up to the third generation, associated with hypomethylation of the *Gilz* promoter, which could be a potential candidate gene that is epigenetically regulated and involved in perinatal programming of cardiovascular diseases across generations.

## Supplementary information

Supplemental Figures s1 and s2
