# Supplementary material for: Preterm birth is associated with epigenetic programming of transgenerational hypertension in mice
Source: Exp Mol Med. 2020 Jan 24;52(1):152–65. doi: 10.1038/s12276-020-0373-5 (PMC7000670; doi:10.1038/s12276-020-0373-5)
Supplement: Supplementary file 1 — Supplemental Figures s1 and s2 [file 12276_2020_373_MOESM1_ESM.pdf]

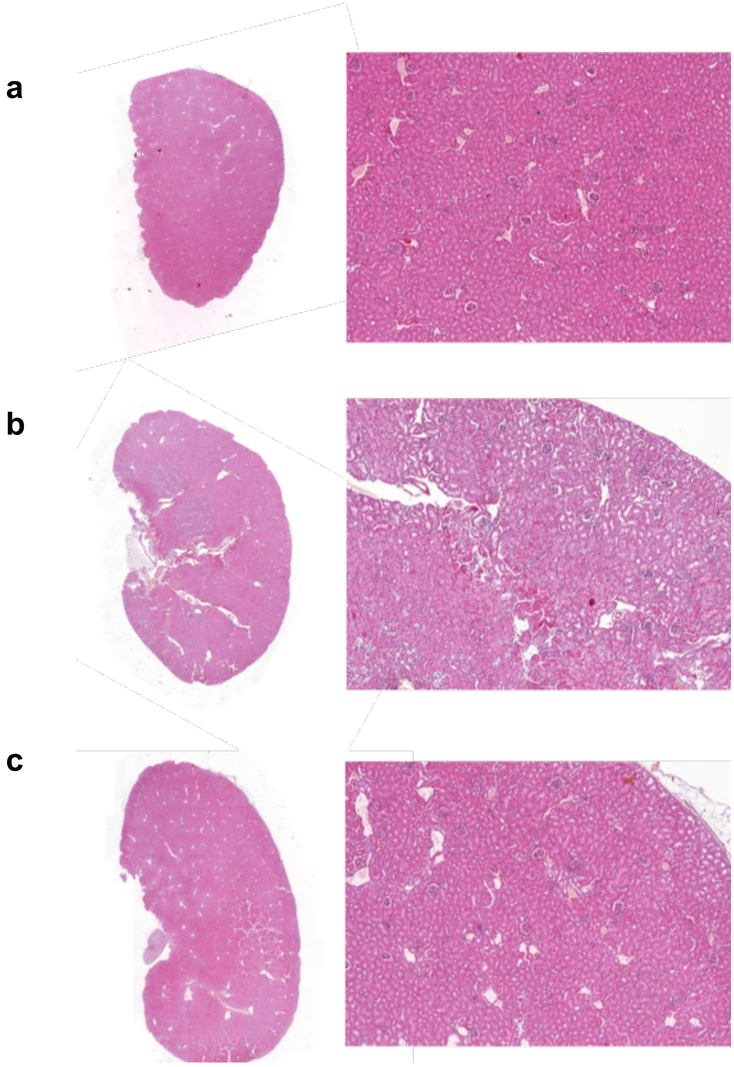

Supplemental Figure s1 : Histologic section of adult kidney from PBS control (**a**), LPS control (**b**) and preterm group (**c**), performed for a manual, double-blind, nephron number quantification. Renal sections were hematoxylin-eosin stained and scanned. The kidneys are presented with a magnification at x0.9 on the left, and x5.6 on the right.

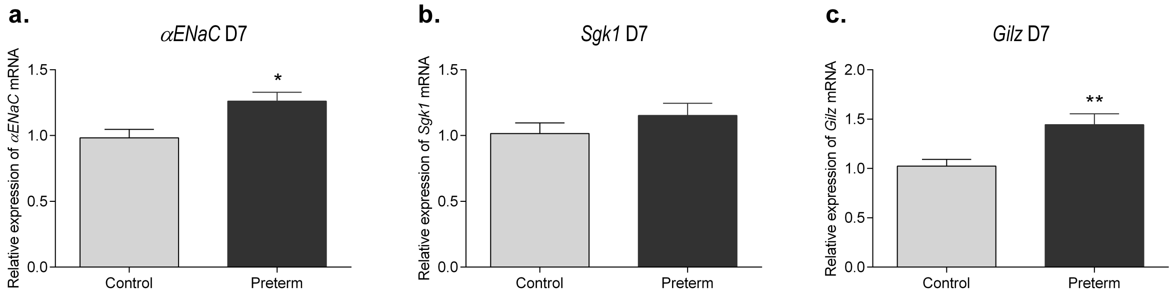

Supplemental Figure s2 : Relative renal expression of αENaC (**a**), Sgk1 (**b**) and Gilz (**c**) mRNA at D7 in control and preterm mice of the F1 generation, determined using reverse transcription-quantitative PCR (RT-qPCR), n=6 mice in each group, * P<0.05, ** P<0.01, *** P<0.001.
